# Adaptive Image Enhancement Algorithm Based on Variable Step Fruit Fly Optimization Algorithm and Nonlinear Beta Transform

**DOI:** 10.3390/biomimetics8020212

**Published:** 2023-05-22

**Authors:** Huajuan Huang, Dao Tao, Xiuxi Wei, Yongquan Zhou

**Affiliations:** 1 College of Artificial Intelligence, Guangxi Minzu University, Nanning 530006, China; 2 College of Electronic Information, Guangxi Minzu University, Nanning 530006, China

**Keywords:** fruit fly optimization algorithm, variable step size, function test, beta transform, image enhancement

## Abstract

Due to the traditional use of manual methods for the parameter adjustment of a nonlinear beta transform, which is inefficient and unstable, an adaptive image enhancement algorithm based on a variable step size fruit fly optimization algorithm and a nonlinear beta transform is proposed. Utilizing the intelligent optimization characteristics of the fruit fly algorithm, we automatically optimize the adjustment parameters of a nonlinear beta transform to achieve better image enhancement effects. Firstly, the dynamic step size mechanism is introduced into the fruit fly optimization algorithm (FOA) to obtain a variable step size fruit fly optimization algorithm (VFOA). Then, with the adjustment parameters of the nonlinear beta transform as the optimization object, and the gray variance of the image as the fitness function, an adaptive image enhancement algorithm (VFOA-Beta) is obtained by combining the improved fruit fly optimization algorithm with the nonlinear beta function. Finally, nine sets of photos were used to test the VFOA-Beta algorithm, while seven other algorithms were used for comparative experiments. The test results show that the VFOA-Beta algorithm can significantly enhance images and achieve better visual effects, which has a certain practical application value.

## 1. Introduction

With the advent of the information age, types of information have become more and more abundant and diverse. Videos, pictures, audios and text are the main information dissemination carriers, while images are the basis for the human perception of the world and the main way to receive and transmit digital information [1]. Due to the influence of factors such as sensors and weather, the image is prone to the problems of sharpness, contrast and decreases in brightness, which seriously affect subsequent information extraction and pattern recognition. It is necessary to enhance the image to improve the image quality. In recent years, many scholars have proposed related image enhancement methods and successfully applied them to practical engineering fields.

Pan Qiang and Yin Jian proposed an image enhancement algorithm based on weight constraint decisions. By establishing a gray histogram equalization model, the image is given fusion weight factors to achieve the purpose of image enhancement [2]. Himanshu Singh, Anil Kumar, et al. proposed a dark image enhancement method based on swarm intelligence optimization and segmented gamma correction. Using the characteristics of gamma correction and histogram equalization, they proposed a weighted summation framework. Additionally, the swarm intelligence algorithm has been used to optimize the fitness function, and the image is enhanced by optimizing the adjustment parameters [3]. Tian Huijuan, Cai Minpeng and others conducted a study on a Retinex low illumination image enhancement method based on YCbCr color space. The Retinex model and multi-scale detail method are used to enhance the image in order to obtain better image quality [4]. Kin et al. [5] conducted research on issues related to low-light networks (LLNet networks), and the experimental results showed that LLNet networks can be applied to processing low-light medical images. The proposal of this idea marks the beginning of deep learning models in medical image enhancement processing with a practical significance. At the same time, Jiang Tao [6] integrated traditional image feature fusion technology and, based on this, utilized convolutional sparse encoding and image enhancement methods to combine automatic learning and the extraction of nodule features. He also developed an automatic recognition algorithm for CT abdominal lymph node detection in order to greatly improve the accuracy of nodule classification. Song et al. [7] conducted research on the related issues of multi task cascaded CNN framework image enhancement, and the research results showed that this method can be used to detect information in thyroid ultrasound images, while achieving automatic detection and recognition of thyroid nodules, resulting in a detection accuracy of up to 98.2%. Lqbal et al. [8] proposed using the MI-GAN network image enhancement method to generate retinal images and supervised and classified them. Abhishck et al. [9] studied the enhancement of the original skin cancer ISIC2017 dataset based on GAN networks and trained the Mask2sion model via segmentation masks, resulting in an accuracy improvement of 5.17% in segmentation testing. Liao Shimin et al. [10] proposed an improved low-dose CT image enhancement network based on CycleGAN. This network enhances the extraction ability of CT image features by adding a shallow feature preprocessing module in front of the generator, further achieving image enhancement. Neha Singh and Ashish Kumar Bhandari [11] proposed a new nighttime input image enhancement algorithm based on multi-scale reflection components. This model not only restores the contrast of the image but also highlights the hidden details in the input while preserving the natural colors in the image. Qingliang Jiao, Ming Liu and Bu Ning, et al. [12], in order to improve the quality of image defogging, proposed a new defogging model based on the fractional derivative and data-driven regularization term. A large number of experimental results show that this method has a certain degree of progressiveness.

The above image enhancement methods have achieved good results in their respective fields, but these image enhancement methods are generally only developed for a specific problem, which has obvious limitations and is not conducive to promotion and use. In addition, each method requires a complex modeling process, which is time-consuming and laborious. Tubbs proposed a normalized nonlinear beta function in 1987 for fitting nonlinear transformation curves. In terms of image enhancement, the nonlinear Beta transform has a very good effect. However, the image enhancement effect of the nonlinear Beta transform depends on the adjustment parameters. The traditional parameter adjustment of the nonlinear Beta function mostly adopts the manual method and the exhaustive method, and the image is enhanced by manually adjusting the parameters. This method has a certain blindness and a lack of intelligence.

Swarm intelligence optimization algorithm has been developed over the past 20 years. It is widely applied for optimization problems, such as imbalanced data classification [13], research on the dynamic reconfiguration of power grids [14], medical insurance intelligent auditing systems [15] and edge detection of mechanical parts images [16]. Its successful application in these fields shows that this is a very effective method. The swarm intelligence optimization algorithm has the main characteristics of intelligence and parallel search. At the same time, it also has the basic characteristics of asymptotic optimization, guided random search, and global optimal solution, and it is easy to combine with other algorithms. As one of the swarm intelligence optimization algorithms, the FOA also has the above advantages. In addition, it also has the characteristics of less adjustment parameters, a fast running speed and ease of use. Therefore, in view of the shortcomings of nonlinear Beta transformation, this paper proposes a variable step-based fruit fly algorithm (VFOA) to optimize the nonlinear Beta function model. First, a variable step size mechanism is introduced to dynamically adjust the optimization step size of the fruit fly algorithm, balance the global search and local optimization capabilities of the algorithm and improve the performance of the algorithm. Then, the VFOA is used to optimize the adjustment parameters of the nonlinear Beta function, and the VFOA-Beta image adaptive enhancement algorithm is established. The VFOA-Beta algorithm can automatically optimize the adjustment parameters of the Beta function, avoiding the disorder and blindness of manual adjustment. Due to the introduction of the FOA algorithm, the image enhancement method of nonlinear Beta transformation has a certain degree of intelligence, which improves the efficiency of image enhancement. The image enhancement method mentioned above does not have the ability of intelligent adjustment, and the modeling process is relatively complicated, which is not conducive to popularization and use. The VFOA-Beta algorithm is relatively simple and easy to use, it can automatically enhance the image without manually adjusting the parameters. Finally, the proposed enhancement algorithm is compared and tested by using eight groups of image data. The experimental results show that, compared to other algorithms, the VFOA-Beta algorithm has a better image enhancement effect, which proves the effectiveness of the improved algorithm.

The structure of this paper is organized as follows: The preliminaries of FOA and VFOA are introduced in Section 1. In Section 2, the proposed VFOA approach is tested on six benchmark functions. Section 3 introduces the traditional image enhancement methods and demonstrates the design of the proposed VFOA-Beta in detail. The performance and effectiveness of VFOA-Beta are verified by six group photos in Section 4. Finally, Section 5 provides some conclusions and future works.

## 2. Improvement Algorithm

### 2.1. Fruit Fly Optimization Algorithm

The FOA was proposed by Wenchao Pan [17], a Ph.D. from Huaxia University of Science and Technology, Taiwan, in 2012. It is a new method to seek global optimization based on the foraging behavior of fruit flies. The optimization mechanism of the algorithm is mainly divided into two parts: first, the fruit fly population uses an olfactory search to approach the optimal solution in the solution space, and, then, other fruit flies use a visual search to find the optimal individual and fly to this direction. This is repeated until the optimal solution is found. 

The basic steps of the FOA are as follows:

Step 1: Initialize the position of the fruit fly population randomly.

(1) $$Init\;X_{-axis}\;,\;Init\;Y_{-axis}$$

Step 2: A fruit fly conducts random (olfactory) searches to generate new locations.

(2) $$\left\{\begin{array}{c} X_{i}=X_{-axis}+Random\;Value\\ Y_{i}=Y_{-axis}+Random\;Value \end{array}\right.$$

Step 3: Calculate the distance between the individual fruit fly and the origin and then obtain the taste judgment value $S_{i}$

(3) Disti=sqrt(Xi^2+Yi^2)

(4) $$S_{i}=1/Disti$$

Step 4: Substitute the taste judgment value into the judgment function to acquire the fitness (taste concentration) value of the fruit fly.

(5) $$Smell(i)=Function(S_{i})$$

Step 5: Retain optimal fitness fruit fly.

(6) [ bestSmell  bestIndex ]=min(Smell)

Step 6: Record the fitness value and position of the best individual. Then, all flies fly to this position using a visual search.

(7) Smellbest=bestSmell

(8) $$\left\{\begin{array}{c} X_{-axis}=X(bestIndex)\\ Y_{-axis}=Y(bestIndex) \end{array}\right.$$

Step 7: In the case of iterative operations, repeat steps 2 to 6; when the maximum iterative value is reached, the optimal fruit fly individual is output.

### 2.2. VFOA Algorithm

The basic fruit fly optimization algorithm has the advantages of a simple structure, few control parameters, and ease of use, and its running speed is extremely fast. However, the FOA also has similar issues to other swarm intelligence algorithms. Due to the random search step size used in the iterative optimization process of the FOA algorithm, the optimization process is blind and disorderly, and the search range is small, resulting in low optimization accuracy and local optimal solutions that are easy to fall into. In response to this deficiency, a dynamic search step size is introduced to improve the optimization process of the FOA, utilizing the ordered convergence characteristics of the improved function to improve the solving efficiency of the original algorithm and balance the global search and local optimization capabilities of the algorithm. The improvement formula is as follows:

(9) $$l_{v}=e^{i/gen}-w*i*e^{-i/gen}$$

**Figure 1:**
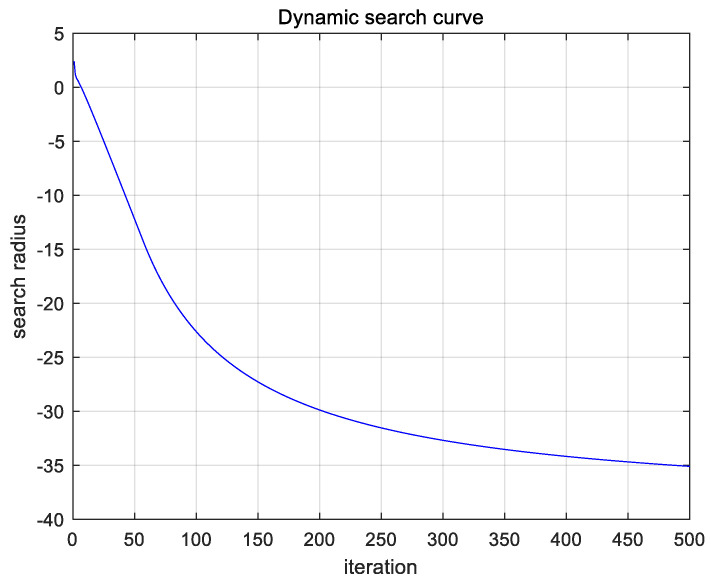
Search step variation.

In this formula, i represents the current fruit fly individual, gen represents the current number of iterations, and w is a weight factor of 0 to 1. In order to facilitate the description of the search curve, each generation of the search step takes the minimum value. When the weight factor is set to 0.8, the population size is 50, and the maximum number of iterations is 500. The change curve of the search step size $l_{v}$ is shown in Figure 1.

As shown in Figure 1, the variable step size mechanism improves the range of search step size changes in the original algorithm, greatly expanding the effective search space of the algorithm and improving the diversity of solutions generated by the original algorithm. In addition, the improved algorithm’s search step size can achieve a convergence adjustment with the increase in iteration times, making the algorithm’s solving process more efficient and orderly, solving the shortcomings of the original algorithm’s random search step size, and effectively improving the algorithm’s optimization efficiency.

## 3. Function Test

### 3.1. Parameter Settings and Experimental Evaluation Criteria

In order to verify the effectiveness of the algorithm’s improvement and test the optimization performance of the VFOA, six benchmark functions were selected for the simulation test to find the minimum value in this paper. Concurrently, the FOA, the chicken swarm algorithm (CSO), the bat algorithm (BA) and the flower pollination algorithm (FPA) were used for comparative experiments. The test functions are shown in Table 1.

In the experiment, each algorithm runs independently 30 times, the population size is 50, and the termination condition is set as the number of iterations reaches 500. All algorithms are programmed with Matlab R2017b, and the computer configuration is as follows: Intel Core(TM) i5-6300hq; 2.3 GHz; 8 GB memory; Windows 10 operating system.

In order to evaluate the optimization effect of the algorithm, this paper gives the following four judgment criteria: (1) the average value (mean), which is the expectation of the optimal value obtained after the algorithm runs 30 times and is used to measure the average quality of the algorithm optimization; (2) the standard deviation (std) between the optimal value and the average optimal value obtained after the algorithm runs 30 times, which is used to evaluate the optimization stability of the algorithm; (3) the optimal value (best), which is the global optimal solution obtained by running the algorithm 30 times; (4) the worst value (worst), which is the global worst solution obtained by running the algorithm 30 times.

### 3.2. Experimental Results and Analysis

For the six standard test functions in Table 1, each algorithm is independently tested 30 times. The evaluation indicators are the best value (best), the worst value (worst), the average value (mean) and the standard deviation (std); the comparison algorithms are VFOA, FOA, CSO, TLBO and FPA in turn. The test comparison results are shown in Table 2.

According to the test data in the above table, the indicators of the VFOA algorithm are better than the FOA. First, it can be seen that the FOA’s std fluctuates greatly, while the VFOA fluctuates less, which indicates that the VFOA has higher stability. Second, it can be seen from the *best* value that the VFOA algorithm can basically find the optimal value, while the FOA cannot, which indicates that the VFOA has stronger global optimization ability. Finally, when comprehensively comparing the test results of the five algorithms, the indicators of the VFOA algorithm are basically optimal. However, the CSO algorithm also showed good optimization performance in the function tests of *F*_4_ and *F*_5_, but the stability of the original algorithm was not good, and the optimization performance of the algorithm fluctuated greatly. In summary, the addition of the variable step size mechanism makes the optimization process of the algorithm more efficient and stable, improves the optimization efficiency of the FOA and verifies the effectiveness of the VFOA.

## 4. Image Nonlinear Enhancement

### 4.1. Traditional Nonlinear Transformation

The basic expression of the image pixel grayscale transformation is as follows:

(10) $$I_{\mathrm{xy}}^{*}=f(i_{xy})$$

**Figure 2:**
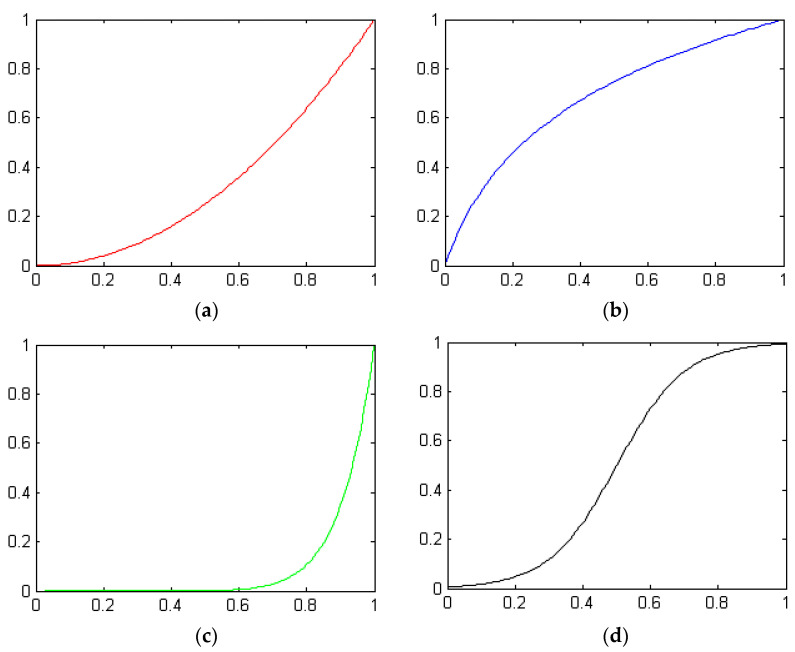
Four typical gray transformation functions. (**a**) Square transformation; (**b**) Logarithmic transformation; (**c**) Gamma transformation; (**d**) Sigmoid transformation.

In the formula, *i_xy_* is the gray value of the pixel point (*x*, *y*) of the original image; I is the gray value of the output-enhanced image pixel point (*x*, *y*); f is the nonlinear transformation. Generally, different transformation functions are used for images of different quality, and there are roughly four types of transformation functions, as shown in Figure 2.

In the above figure, the horizontal axis represents the gray value of the original image, and the vertical axis represents the gray value of the transformed image. (a) The square transformation is mainly used to reduce image brightness, enhance contrast in bright parts and slow down contrast in dark parts. (b) The logarithmic transformation is suitable for improving image brightness, enhancing contrast in dark parts and slowing contrast in bright parts. (c) The role of the gamma transformation is similar to the square transformation, but the image processing effect of the gamma transformation is softer. (d) The sigmoid transformation is mainly used to stretch the middle gray area of the image and compress the gray level of the two end areas.

### 4.2. Nonlinear Beta Transform

The normalized nonlinear Beta transformation function was proposed by Tubbs. This function can automatically fit the four types of transformation curves mentioned in Section 3.1. Its basic definition is as follows:

(11) $$F(u)=B^{-1}(\alpha ,\beta )\int_{0}^{u}t^{\alpha -1}{\left(1-t\right)}^{\beta -1}dt,\;0<\alpha ,\beta <10$$

In Formula (11), B(α,β) is the Beta function, t is the integral variable, and its specific expression is as follows:

(12) $$B(\alpha ,\beta )=\int_{0}^{1}t^{\alpha -1}{\left(1-t\right)}^{\beta -1}dt$$

It can be seen from Equations (11) and (12) that, as long as the parameters α,β are adjusted, the four types of transformation curves in Figure 2 can be automatically fitted. Therefore, the fitting effect of the nonlinear Beta transform function mainly depends on the parameter α,β. According to the characteristics of the nonlinear Beta function, when α<β, dark images can be enhanced; when α>β, bright images can be enhanced. Therefore, the nonlinear Beta transform has better adaptability.

### 4.3. Fitness Function

According to Section 3.2, the image enhancement effect based on the nonlinear Beta transform mainly depends on the selection of parameters. The traditional parameter selection method has strong subjectivity and blindness and cannot deal with the complex image process. Thus, this paper adopts the VFOA algorithm proposed in Section 2.2 to solve the problem of parameter selection and regards the selection of parameters α,β as the process of finding the optimal solution. Taking the variance of the gray value of the image as the optimization function, the larger the variance, the richer the content of the image and the larger the dynamic range of image pixel values. For an image of M∗N, the variance is calculated as follows:

(13) $$FC=\frac{1}{M*N}\sum_{x=1}^{M}\sum_{y=1}^{N}i_{xy}^{2}-(\frac{1}{M*N}\sum_{x=1}^{M}i_{xy}{)}^{2}$$

Since the fruit fly algorithm is to find the minimum value, the fitness function is designed as follows:

(14) fitness=−Fc

In the Formula (13), M and N represent the column and row of the image, respectively, and $i_{xy}$ represents the pixel value of the image. In Formula (14), the variance calculation formula is inverted and converted into the fitness function of the VFOA.

### 4.4. VFOA-Beta Image Enhancement Algorithm

This paper uses the VFOA algorithm proposed in Section 2.2 to determine the α and β parameters in the nonlinear Beta function and then achieves the purpose of image enhancement using the nonlinear Beta function. The specific steps of the algorithm are as follows:

Step1. Determine whether the image is a grayscale one; if not, convert the image to grayscale.

Step2. Normalize the grayscale image. The formula is as follows:

(15) $$n(x,y)=\frac{\left[I(x,y)-g_{\min}\right]}{g_{\max}-g_{\min}}$$

In the formula, I(x,y) represents the gray value of the original image, $g_{\min}$ represents the minimum gray value of the original image, $g_{\max}$ represents the maximum gray value of the original image, n(x,y) represents the normalized image gray value, and n(x,y)∈(0,1).

Step3. Use the VFOA algorithm proposed in Section 2.2 to optimize the adjustment parameters of the nonlinear Beta function.

Step4. Substitute the calculated optimal parameters into Equation (11) to construct a specific nonlinear Beta transformation function F(x).

Step5. Use the nonlinear Beta transform function to process the normalized image gray value.

(16) $$n^{\prime }(x,y)=F(n(x,y))$$

Step6. Denormalize the enhanced grayscale image. The formula is as follows:

(17) $$I_{b}=g_{\min}+n^{\prime }(x,y)(g_{\max}-g_{\min})$$

## 5. Experimental Analysis

### 5.1. Algorithm Parameter Settings and Experimental Results

In order to verify the effectiveness of the VFOA-Beta algorithm, eight sets of images are selected for testing in this paper. At the same time, the FOA-Beta, SSA-Beta (sparrow search algorithm), CSO-Beta (chicken swarm optimization), MFO-Beta (moth–flame optimization), FPA-Beta (flower pollination algorithm), HE (histogram equalization) and RETINEX algorithms are used to conduct comparative experiments to test the performance of the improved algorithm. The parameters of the VFOA-Beta algorithm are set to 30 populations, 50 iterations, and a weight factor of 0.8; the main parameter settings of the FOA-Beta and SSA-Beta algorithms are the same as the VFOA-Beta. The experimental environment is the same as before.

**Figure 3:**
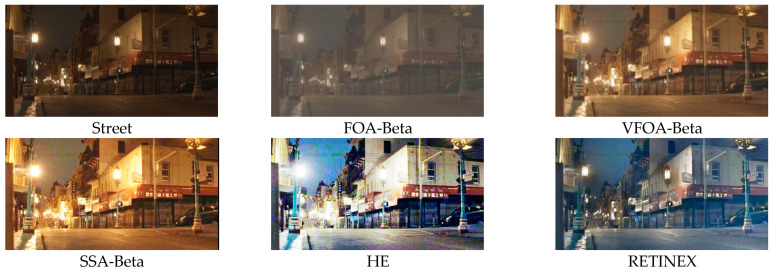

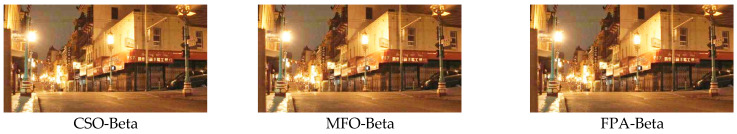
Street diagram and its enhancement diagram.

**Figure 4:**
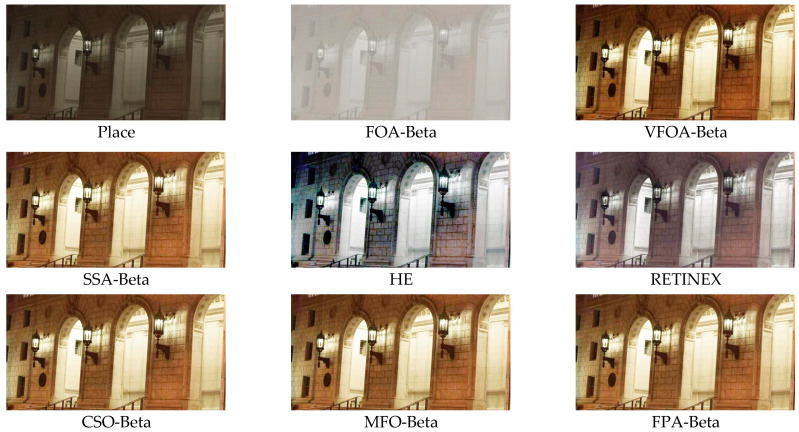
Place diagram and its enhancement diagram.

**Figure 5:**
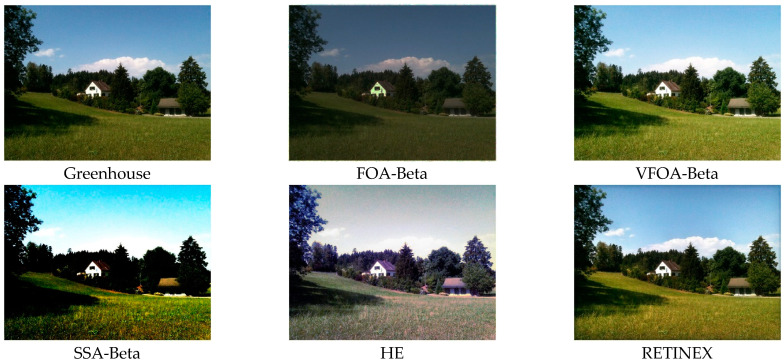

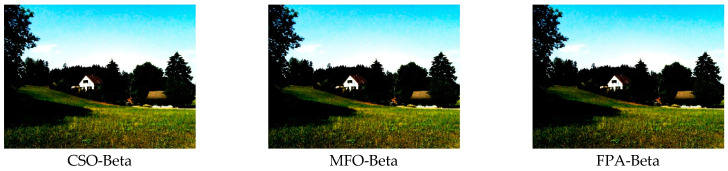
Greenhouse diagram and its enhancement diagram.

**Figure 6:**
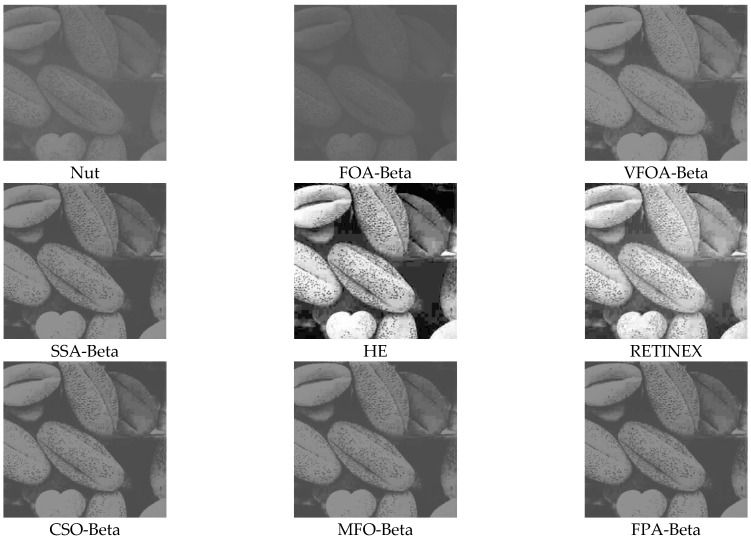
Nut diagram and its enhancement diagram.

**Figure 7:**
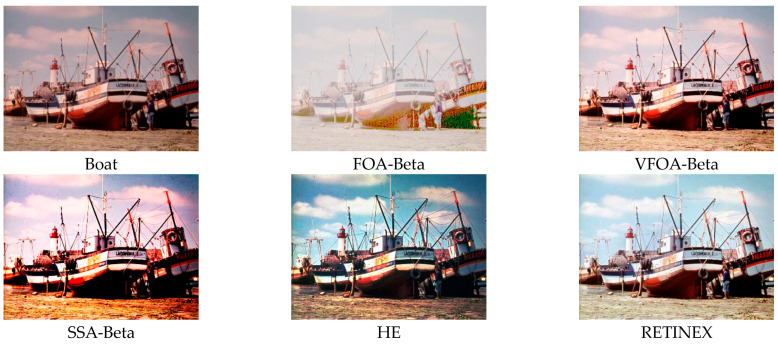

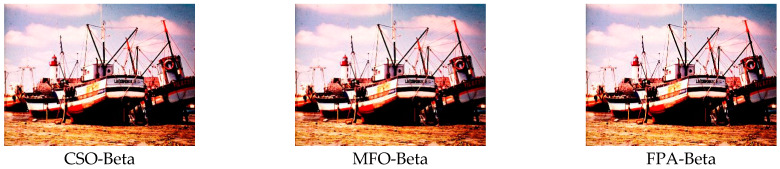
Boat diagram and its enhancement diagram.

**Figure 8:**
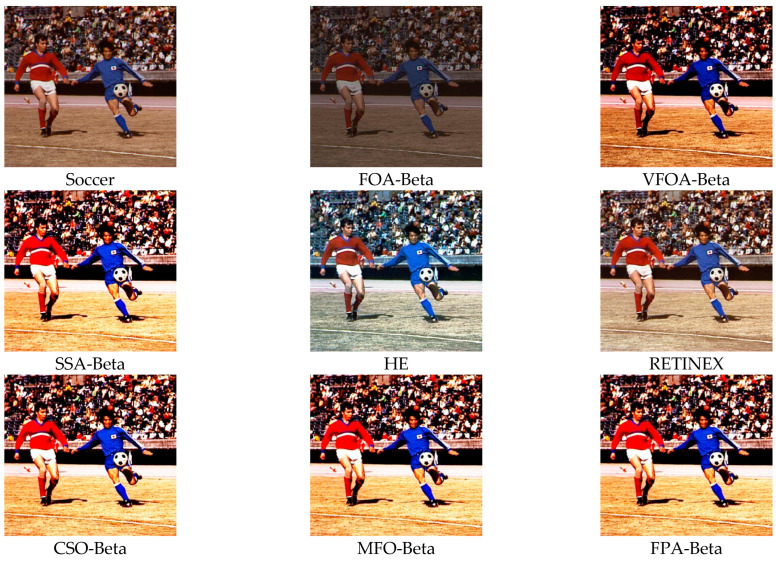
Soccer diagram and its enhancement diagram.

**Figure 9:**
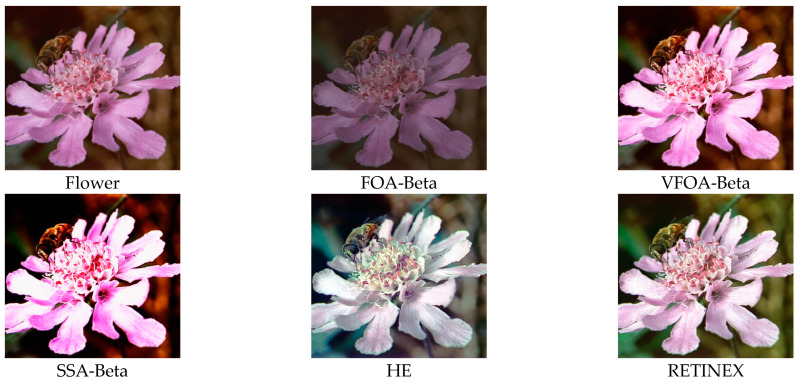

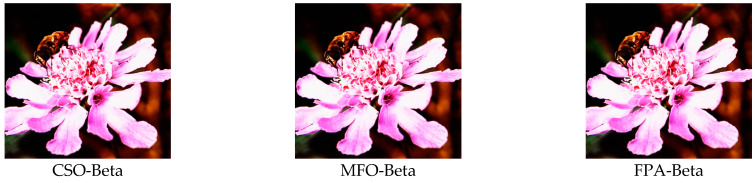
Flower diagram and its enhancement diagram.

**Figure 10:**
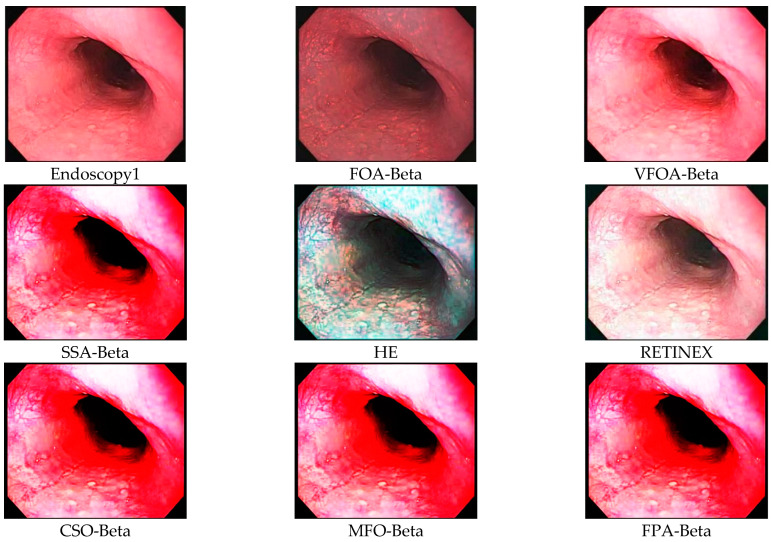
Endoscopy1 diagram and its enhancement diagram.

**Figure 11:**
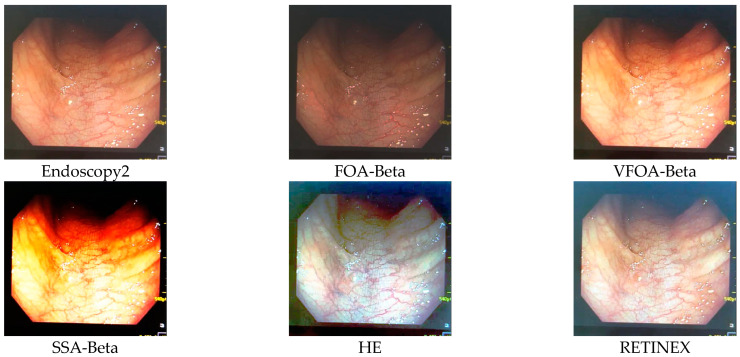

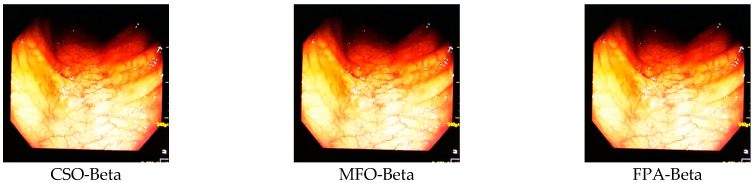
Endoscopy2 diagram and its enhancement diagram.

This section selects six representative images, named Street, Place, Greenhouse, Nut, Lena and Boat. In order to avoid the chance of the experiment, this paper selects pictures displaying different scenarios for comparative testing. The image enhancement effects are shown in Figure 3, Figure 4, Figure 5, Figure 6, Figure 7, Figure 8, Figure 9, Figure 10 and Figure 11. From left to right are the original image, the FOA-Beta-enhanced image, the VFOA-Beta-enhanced image, the SSA-Beta-enhanced image, the HE-enhanced image, and the RETINEX-enhanced image.

After the test and comparison of the above nine groups of pictures, it can be found that the image enhancement effect of the VFOA-Beta algorithm is significantly better than the FOA-Beta algorithm. The image enhanced by the FOA-Beta algorithm is generally white, and the image enhancement effect is not significant for the Street, Greenhouse, Nut and remaining images. The SSA-Beta algorithm, CSO-Beta algorithm, MFO-Beta algorithm and FPA-Beta algorithm also have good enhancement effects on the images. In Figure 3 and Figure 4, these algorithms outperform VFOA-beta, but they are prone to excessive enhancement. For example, it can be found in the Boat enhancement image that the color of the sea water is too bright red, and the Greenhouse, Flower, Endoscopy1 and Endoscopy2 images show similar problems; The enhancement of the image by the HE method is generally prone to the phenomenon of image color distortion. For example, the color of the leaves in the Greenhouse picture and the color of the endoscope wall in the Endoscopy1 picture have a large color deviation from the original picture. However, the HE method has a good performance in the grayscale image Nut, which greatly improves the clarity of the original image. The RETINEX algorithm has a good overall image enhancement effect, as shown in the image enhancement and restoration effect of Greenhouse and Nut. However, the RETINEX algorithm also suffers from distortion. For example, in the Street enhancement map, the color of the wall and the streetlights are relatively cool. Overall, the image enhanced by the VFOA-Beta algorithm has better contrast and clarity, improving the overall quality of the image effectively and proving the effectiveness of the VFOA-Beta.

### 5.2. Image Grayscale Analysis

To evaluate the image enhancement performance of the VFOA-Beta algorithm more intuitively, this section will analyze the gray value distribution before and after image enhancement. Due to space limitations, this paper only shows the grayscale distribution of the VFOA-Beta enhanced image and the original image. The grayscale distribution of the image is shown as Figure 12, Figure 13, Figure 14, Figure 15, Figure 16, Figure 17, Figure 18, Figure 19, Figure 20, Figure 21, Figure 22, Figure 23, Figure 24, Figure 25, Figure 26, Figure 27, Figure 28 and Figure 29. From top to bottom are the grayscale distribution of the original image and the VFOA-Beta-enhanced grayscale distribution of the image.

It can be seen from the above figures that the distribution of the image enhanced by the VFOA-Beta algorithm has decreased in the low gray area and increased in the high gray value area, making the gray distribution of the image more extensive and uniform. This shows that the enhanced image has a higher brightness, better color richness and better visual effect. Therefore, the VFOA-Beta algorithm has a better image enhancement effect, which proves that the improved algorithm has a certain practical application value.

### 5.3. Image Quality Analysis

Next, this paper will introduce two important indicators of image quality evaluation, peak signal-to-noise ratio (PSNR) and the structural similarity index measure (SSIM), so as to further analyze the quality of images enhanced by each algorithm objectively. The specific formula is as follows:

(18) $$MSE=\frac{1}{M\times N}\sum_{x=0}^{M}\sum_{y=0}^{N}{\left(I(x,y)-I_{e}(x,y)\right)}^{2}$$

(19) $$PSNR=10\times \log_{10}\left(\frac{MAX_{I}^{2}}{MSE}\right)$$

In the above formula, M and N represent the row and column of the image, respectively, I(x,y) is the pixel value of the original image, $I_{m}(x,y)$ is the pixel value of the enhanced image, and MAX is the maximum value of the image point color. The value of MSE is smaller, and the value of PSNR is higher, which means improved image quality.

SSIM is mainly evaluated from three parameters of image brightness, contrast and structure. The specific formula is as follows:

(20) $$SSIM(a,b)=\frac{\left(2u_{a}u_{b}+c_{1})(2\delta_{ab}+c_{2}\right)}{\left(u_{a}^{2}+u_{b}^{2}+c_{1})(\delta_{a}^{2}+\delta_{b}^{2}+c_{2}\right)}$$

In the above formula, *a* and *b* represent the original image and the enhanced image, respectively; $u_{a}$ represents the mean of *a*; $u_{b}$ represents the mean of *b*; $\delta_{a}^{2}$ and $\delta_{b}^{2}$ represent the variance of *a* and the variance of *b*, respectively; and $\delta_{ab}^{2}$ represents the covariance of *a* and *b*. $c_{1}={\left(k_{1}L\right)}^{2}$, $c_{2}={\left(k_{2}L\right)}^{2}$, L is the dynamic range of pixel values, $k_{1}=0.01$, $k_{2}=0.03$. The value range of SSIM is $\left[0,\;1\right]$; if the value is closer to 1, the distortion rate of the image is smaller.

NIQE: Natural image quality assessment, which is a no-reference image objective evaluation index for quality assessment using image features extracted from images. The smaller the value, the better the image enhancement. The expression looks like this:

(21) $$D(v_{1},v_{2},\sum_{1},\sum_{2})=\sqrt{{\left(v_{1}-v_{2}\right)}^{T}(\frac{\sum_{1}+\sum_{2}}{2})(v_{1}-v_{2})}$$

In the above formula, $v_{1}$, $v_{2}$, $\sum_{1}$ and $\sum_{2}$ represent the model mean and variance matrix of the original image and the distorted image, respectively.

BRISQUE: a non- reference spatial domain image quality evaluation algorithm. The overall principle of the algorithm is to extract mean subtracted contrast normalized (MSCN) coefficients from the image, fit the MSCN coefficients into an asymmetric generalized Gaussian distribution (AGGD), extract the features of the fitted Gaussian distribution, input them into support vector machine (SVM) for regression, and obtain the evaluation results of image quality. The smaller the value, the better the image quality.

(22) $$\hat{I}(i,j)=\frac{I(i,j)-u(i,j)}{\sigma (i,j)+C}$$

(23) $$u(i,j)=\sum_{k=-K}^{K}\sum_{l=-L}^{L}w_{k,l}I_{k,l}(i,j)$$

(24) $$\sigma (i,j)=\sqrt{{\sum_{k=-K}^{K}\sum_{l=-L}^{L}w_{k,l}(I_{k,l}(i,j)-u(i,j))}^{2}}$$

In the above formula, I(x,y) is the MCSN coefficient, u(x,y) is the result of the Gaussian filter, σ(x,y) is the standard deviation, and C is the constant.

Using Equations (18) to (24), the eight groups of images in Figure 3, Figure 4, Figure 5, Figure 6, Figure 7, Figure 8, Figure 9, Figure 10 and Figure 11 are used for quality evaluation calculation, and the evaluation indexes *PSNR* and *SSIM* are obtained. The specific results are shown in Table 3 below.

From the data in Table 3, it can be seen that the VFOA-Beta algorithm has a good performance for index PSNR, index SSIM, index NIQE and index BRISQUE. Compared with other enhancement algorithms, the test indicators of VFOA-Beta can be ranked in the top position, indicating that the improved algorithm has high stability and can maintain the quality of the image itself while improving the image clarity. The HE method performs poorly on the PSNR and the SSIM, indicating that its image enhancement effect is general. Although the SSA-Beta algorithm and the RETINEX algorithm also have good performance in the PSNR, their SSIM often declines seriously, which makes the enhanced image seriously distorted. In terms of NIQE indicator, each algorithm has its own advantages and disadvantages. In addition, the VFOA-Beta algorithm overall performs well in terms of indicators, indicating that the enhanced image has good quality. In general, the above eight algorithms can enhance the image to a certain extent to achieve the purpose of image enhancement. However, based on the above visual comparison, grayscale analysis and objective index analysis, the VFOA-Beta algorithm has a more stable performance and can effectively enhance the image.

## 6. Conclusions

Due to the need to manually adjust the parameters when the nonlinear Beta transform is used for image enhancement, it is challenging and inefficient to use the Beta transform for image enhancement. In order to make the image enhancement of the nonlinear Beta transform more efficient and intelligent, this paper introduces the VFOA algorithm to improve the shortcomings of the traditional nonlinear Beta transform.

Firstly, a VFOA-Beta model is constructed using the VFOA algorithm and nonlinear Beta transform. Subsequently, the effectiveness of the improved model was tested using nine sets of images in different scenarios, and a comparative test was conducted using the FOA-Beta algorithm, SSA-Beta algorithm, CSO-Beta algorithm, MFO-Beta algorithm, FPA-Beta algorithm, HE method, and RETINEX algorithm. Finally, the experimental results show that the VFOA-Beta algorithm can improve image clarity and contrast while ensuring image quality, verifying the effectiveness of the VFOA-Beta algorithm.

## Figures and Tables

**Figure 12:**
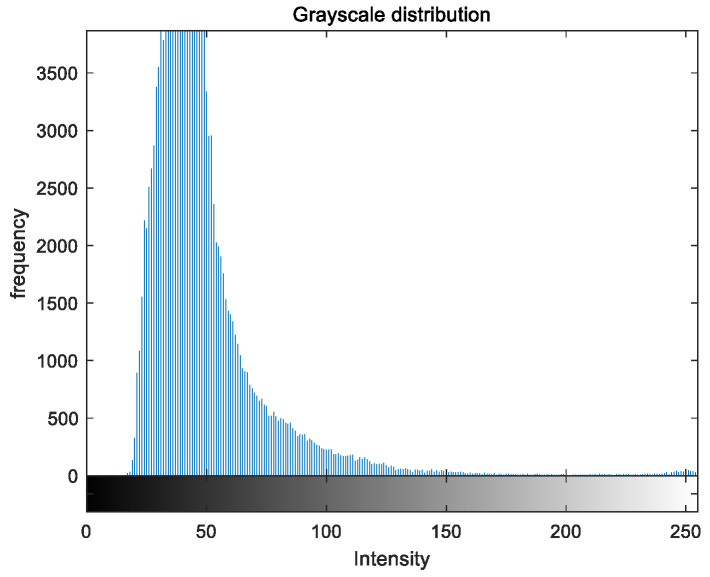
Street gray distribution.

**Figure 13:**
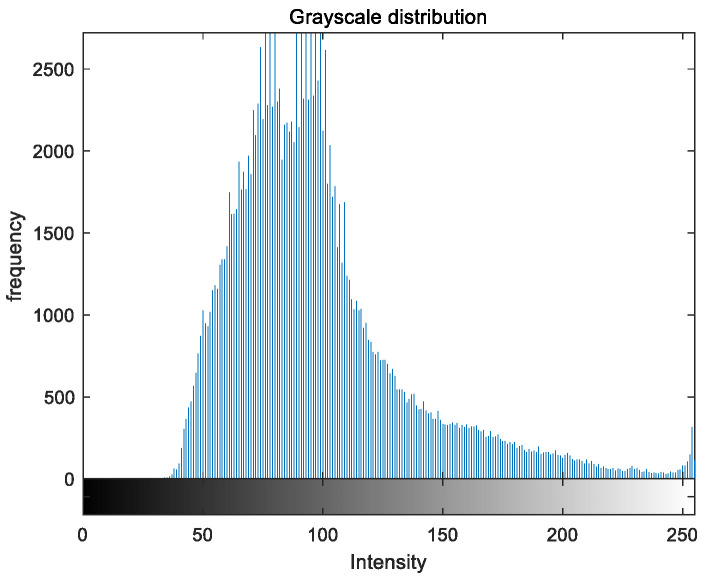
Street enhanced distribution.

**Figure 14:**
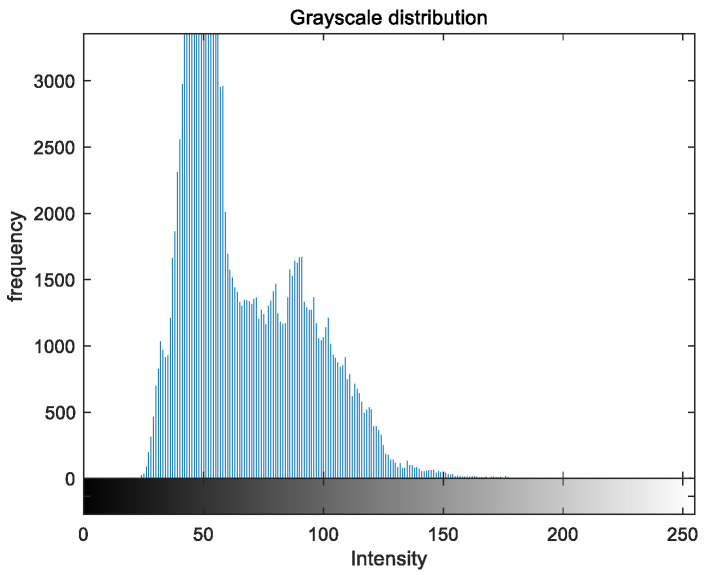
Place gray distribution.

**Figure 15:**
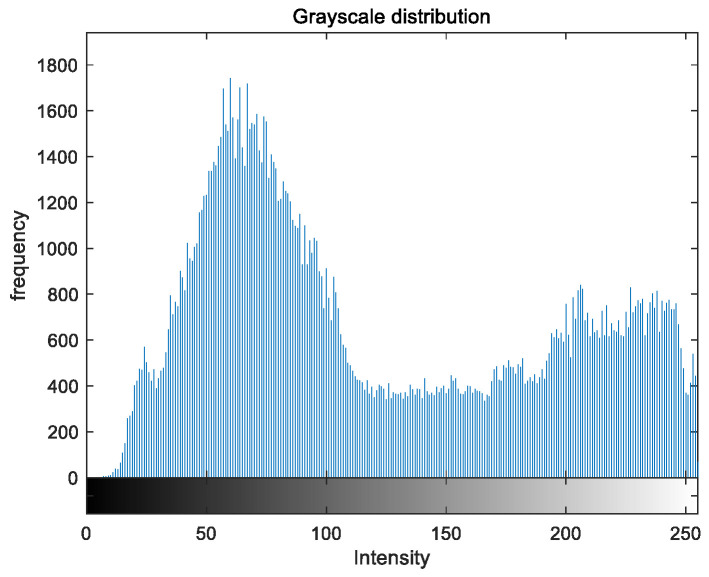
Place enhanced distribution.

**Figure 16:**
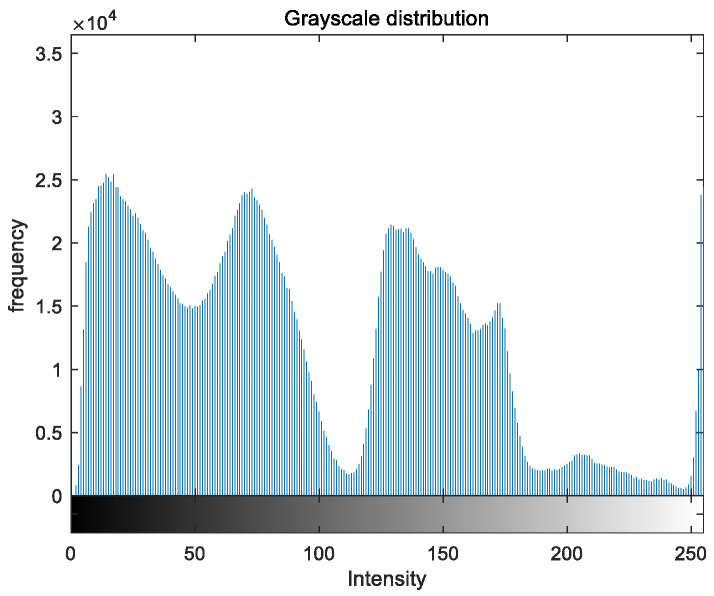
Greenhouse gray distribution.

**Figure 17:**
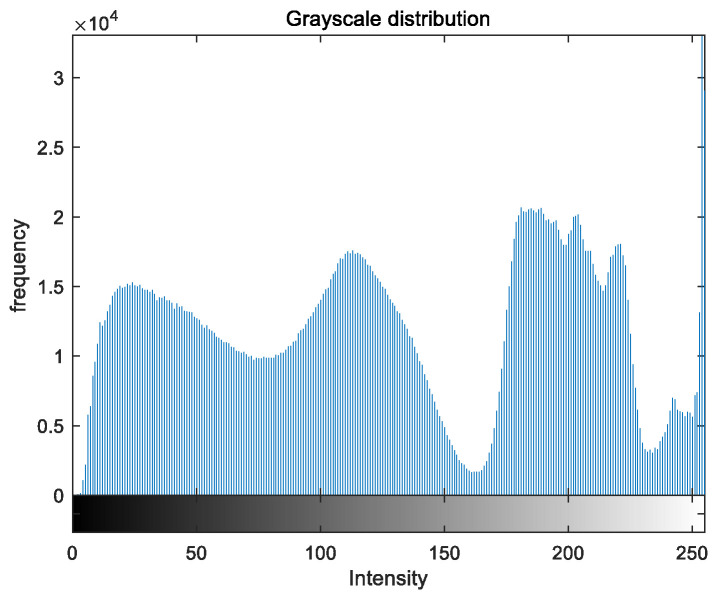
Greenhouse enhanced distribution.

**Figure 18:**
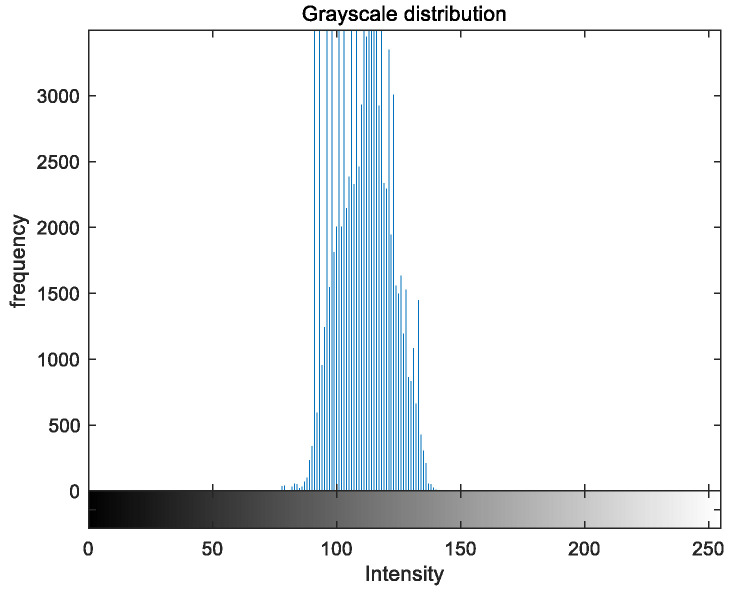
Nut gray distribution.

**Figure 19:**
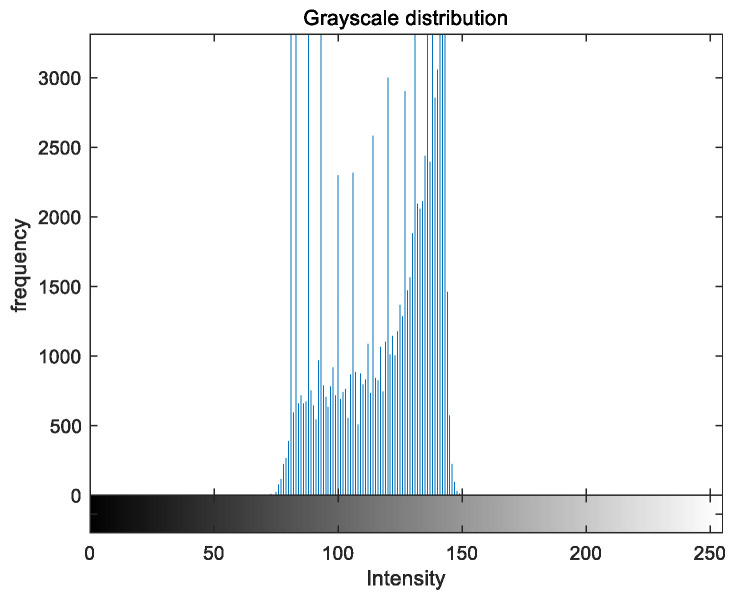
Nut enhanced distribution.

**Figure 20:**
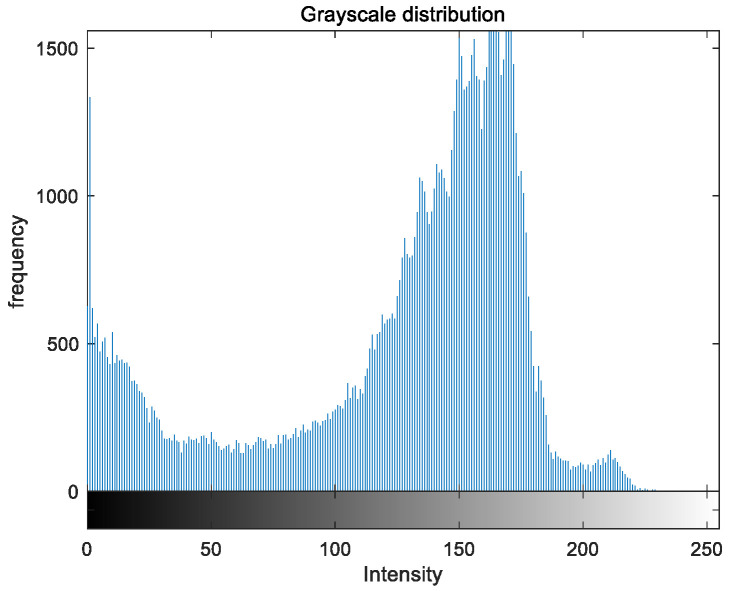
Boat gray distribution.

**Figure 21:**
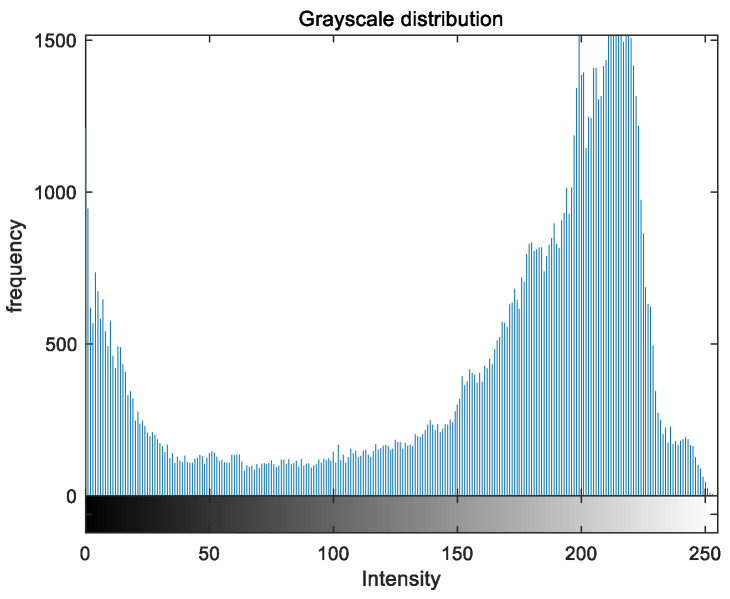
Boat enhanced distribution.

**Figure 22:**
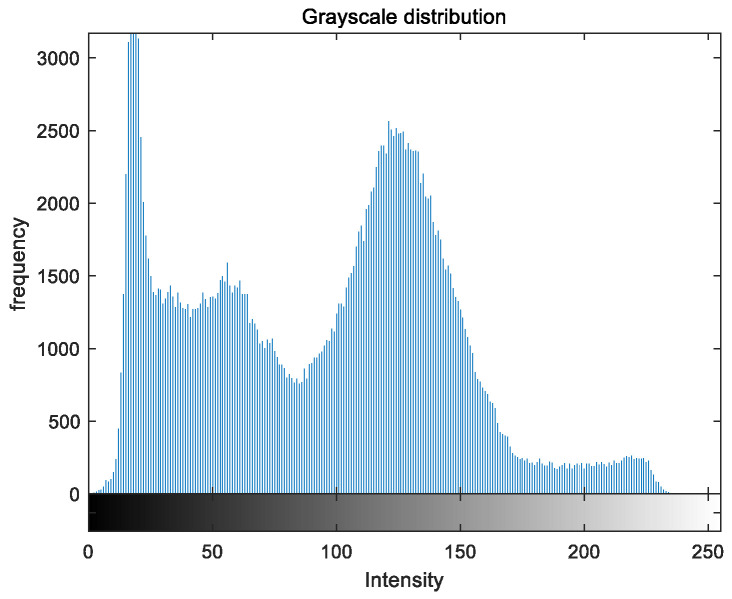
Soccer gray distribution.

**Figure 23:**
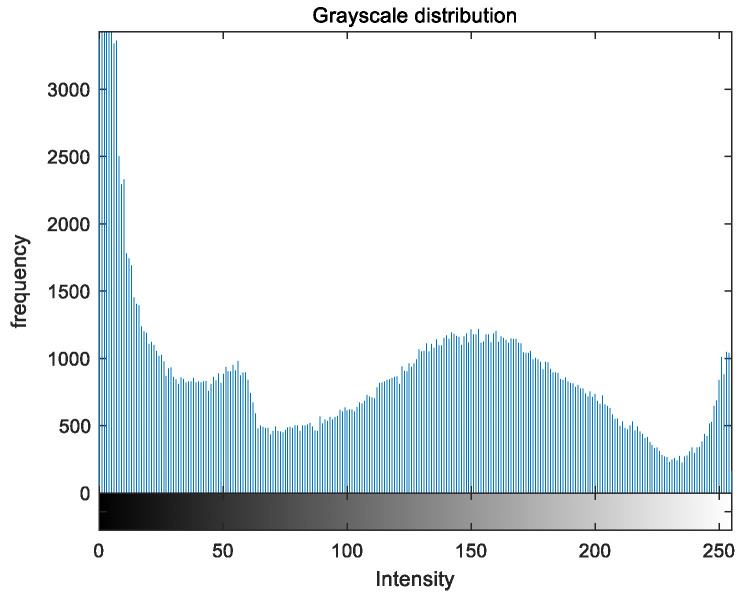
Soccer enhanced distribution.

**Figure 24:**
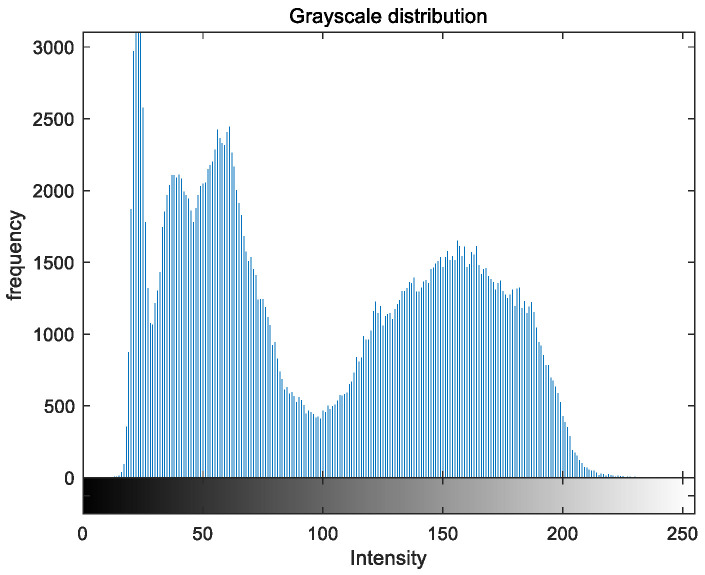
Flower gray distribution.

**Figure 25:**
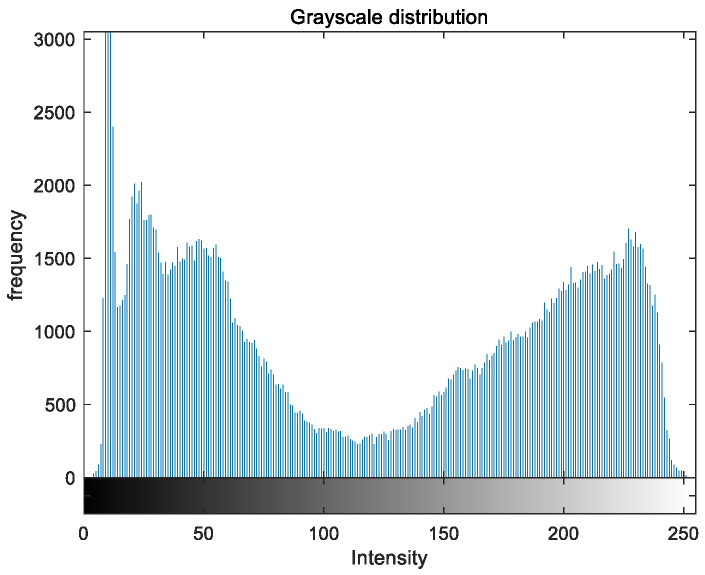
Flower enhanced distribution.

**Figure 26:**
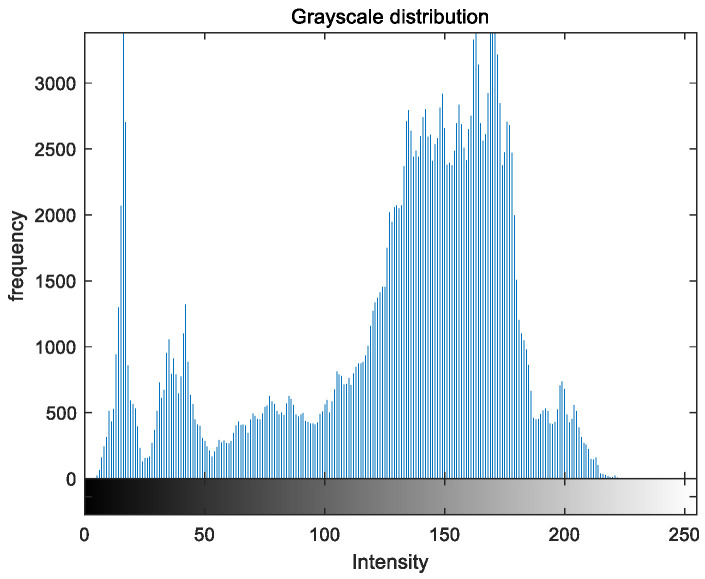
Endoscopy1 gray distribution.

**Figure 27:**
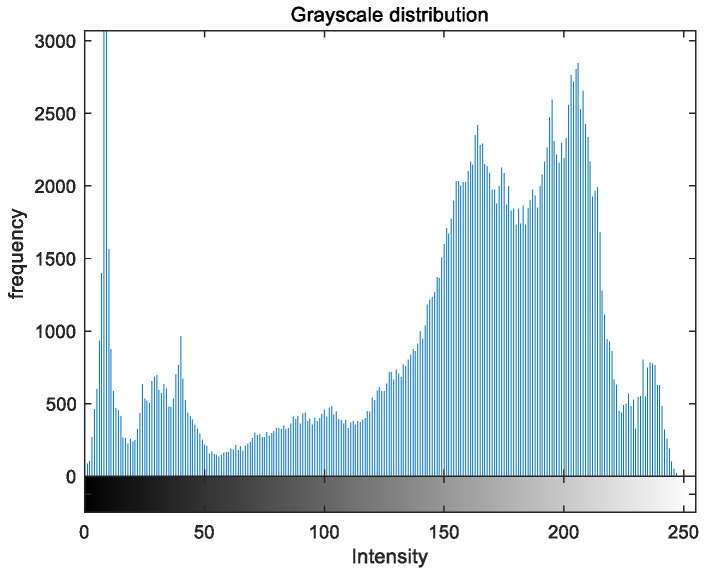
Endoscopy1 enhanced distribution.

**Figure 28:**
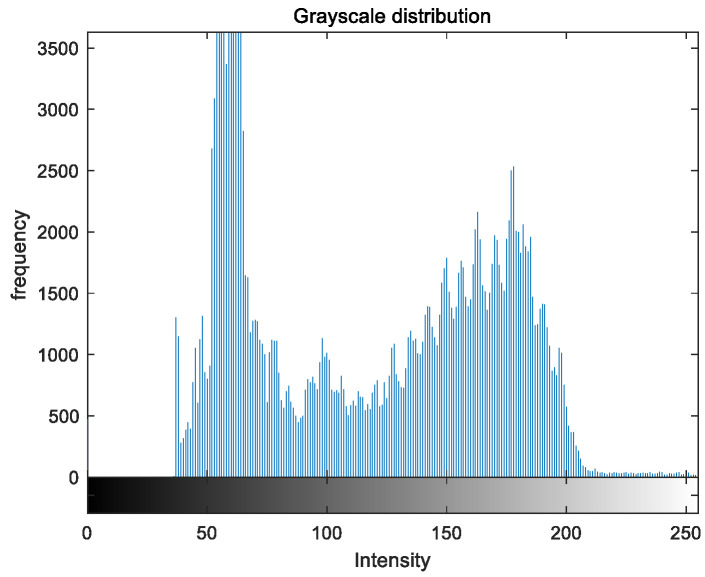
Endoscopy2 gray distribution.

**Figure 29:**
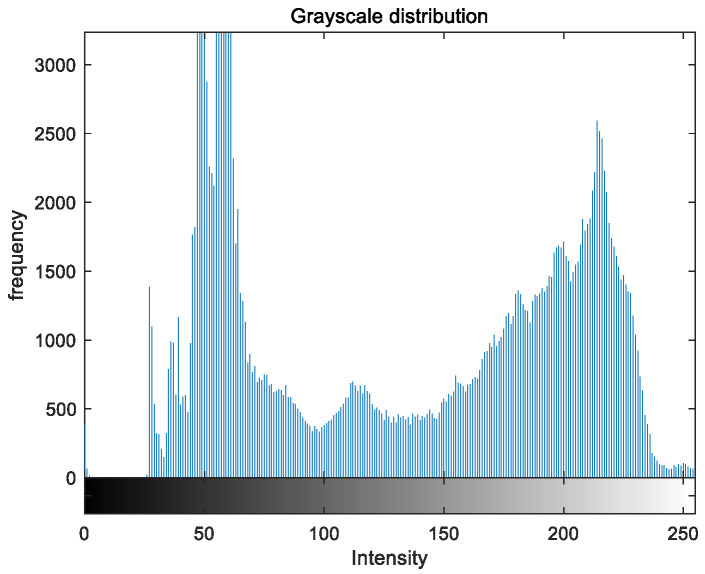
Endoscopy2 enhanced distribution.

**Table 1:** Test functions and parameter setting.

ID	Equation	Bound	Dim	Peak	F (min)
Griewank	$F1\left(x\right)=\sum_{i=1}^{d}\frac{X_{i}^{2}}{4000}-\prod_{i=1}^{d}\cos\left(\frac{x_{i}}{\sqrt{i}}\right)+1$	[−600, 600]	30	multimodal	0
Drop−Wave	$F2(x)=-\frac{1+\cos\left(12\sqrt{x_{1}^{2}+x_{2}^{2}}\right)}{0.5\left(x_{1}^{2}+x_{2}^{2}\right)+2}$	[−5.12, 5.12]	2	multimodal	−1
Rastring	$F3\left(x\right)=10d+\sum_{i=1}^{d}[x_{i}^{2}-10\cos\left(2\pi x_{i}\right)]$	[−5.12, 5.12]	30	multimodal	0
Schaffer N.2	$F4\left(x\right)=0.5+\frac{sin^{2}\left(x_{1}^{2}-x_{2}^{2}\right)-0.5}{{\left[1+0.001\left(x_{1}^{2}+x_{2}^{2}\right)\right]}^{2}}$	[−100, 100]	2	multimodal	0
$\begin{array}{l} Bohachevsky-\\ Rotated \end{array}$	$\begin{array}{l} F_{5.1}\left(x\right)=x_{1}^{2}+2x_{2}^{2}-0.3\cos\left(3\pi x_{1}\right)-0.4\cos\left(4\pi x_{2}\right)+0.7;\\ F_{5.2}\left(x\right)=x_{1}^{2}+2x_{2}^{2}-0.3\cos\left(3\pi x_{1}\right)0.4\cos\left(4\pi x_{2}\right)+0.3;\\ F_{5.3}\left(x\right)=x_{1}^{2}+2x_{2}^{2}-0.3\cos(3\pi x_{1}+4\pi x_{2})+0.3. \end{array}$	[−100, 100]	2	multimodal	0
Matyas	$F6\left(x\right)=0.26(x_{1}^{2}+x_{2}^{2})-0.48x_{1}x_{2}$	[−10, 10]	2	multimodal	0

**Table 2:** Test function results.

Benchmark Function	Method	VFOA	FOA	CSO	BA	FPA
F1	best	0	3.85 × 10^−6^	0	3.93 × 10^−1^	1.70 × 10^1^
	worst	0	1.07 × 10^−5^	1.86 × 10^−3^	6.67 × 10^2^	3.65 × 10^1^
	mean	0	7.38 × 10^−6^	3.72 × 10^−5^	5.65 × 10^2^	2.72 × 10^1^
	std	0	1.55 × 10^−6^	2.63 × 10^−4^	1.19 × 10^2^	5.11
F2	best	−1	−9.36 × 10^−1^	−1	−9.97 × 10^−1^	−9.99 × 10^−1^
	worst	−1	−9.26 × 10^−1^	0	−3.72 × 10^−1^	−9.36 × 10^−1^
	mean	−1	−9.36 × 10^−1^	−2.00 × 10^−2^	−6.80 × 10^−1^	−9.72 × 10^−1^
	std	0	1.93 × 10^−3^	1.41 × 10^−1^	1.59 × 10^−1^	2.39 × 10^−2^
F3	best	0	3.46 × 10^−2^	0	2.48 × 10^2^	1.24 × 10^2^
	worst	0	5.21 × 10^1^	5.07	4.74 × 10^2^	1.89 × 10^2^
	mean	0	3.51 × 10^0^	1.01 × 10^−1^	4.25 × 10^2^	1.53 × 10^2^
	std	0	1.32 × 10^1^	7.17 × 10^−1^	3.90 × 10^1^	1.81 × 10^1^
F4	best	0	4.88 × 10^−9^	0	1.29 × 10^−7^	5.51 × 10^−6^
	worst	0	1.82 × 10^−7^	0	4.61 × 10^−1^	7.56 × 10^−3^
	mean	0	5.88 × 10^−8^	0	2.81 × 10^−1^	1.76 × 10^−3^
	std	0	6.57 × 10^−8^	0	1.27 × 10^−1^	1.76 × 10^−3^
F5	best	0	9.68 × 10^−5^	0	2.93 × 10^−3^	6.30 × 10^−5^
	worst	0	3.82 × 10^−3^	0	1.71 × 10^3^	1.83 × 10^−2^
	mean	0	1.20 × 10^−3^	0	4.24 × 10^2^	3.52 × 10^−3^
	std	0	1.47 × 10^−3^	0	4.55 × 10^2^	3.88 × 10^−3^
F6	best	3.63 × 10^−46^	1.16 × 10^−7^	0	8.01 × 10^−6^	2.21 × 10^−10^
	worst	1.63 × 10^−44^	3.47 × 10^−4^	7.63 × 10^−115^	1.36	2.89 × 10^−8^
	mean	3.01 × 10^−45^	5.23 × 10^−5^	1.53 × 10^−116^	2.60 × 10^−1^	7.40 × 10^−9^
	std	3.78 × 10^−45^	1.19 × 10^−4^	1.08 × 10^−115^	3.29 × 10^−1^	6.94 × 10^−9^

**Table 3:** Enhancement image quality comparison.

Name	Method	*PSNR*	*SSIM*	*NIQE*	*BRISQUE*
Street	VFOA-Beta	14.1233	0.6464	3.1431	25.1340
	FOA-Beta	15.5216	0.7171	3.1920	30.0115
	CSO-Beta	10.9278	0.4334	3.1080	27.8872
	MFO-Beta	10.9232	0.4330	3.1082	28.1446
	FPA-Beta	11.0022	0.4362	3.1947	27.8055
	SSA-Beta	10.9232	0.4330	3.1082	28.1446
	HE	7.9699	0.1983	3.6127	34.5884
	RETINEX	12.4188	0.1118	3.1327	17.6996
Place	VFOA-Beta	11.1564	0.5599	4.2499	35.7580
	FOA-Beta	6.8108	0.4967	3.8923	48.7895
	CSO-Beta	10.2517	0.5287	4.1802	36.3924
	MFO-Beta	10.2512	0.5288	4.1687	36.3923
	FPA-Beta	10.1227	0.5238	4.1717	36.6642
	SSA-Beta	10.2512	0.5288	4.1687	36.3923
	HE	9.6597	0.3290	4.1024	35.2607
	RETINEX	10.7112	0.6247	4.2815	39.1859
Greenhouse	VFOA-Beta	16.7966	0.8703	2.8740	40.3722
	FOA-Beta	15.5845	0.7912	2.9776	41.2553
	CSO-Beta	14.7311	0.5662	2.7852	39.7125
	MFO-Beta	14.7313	0.5667	2.7853	39.7117
	FPA-Beta	14.8927	0.5579	2.8154	39.9711
	SSA-Beta	14.7316	0.5660	2.7848	39.8326
	HE	14.9302	0.4268	2.9486	46.7764
	RETINEX	20.1074	0.9219	2.8942	40.2140
Nut	VFOA-Beta	25.3448	0.9164	6.5643	42.4473
	FOA-Beta	25.7211	0.9547	4.9357	52.5248
	CSO-Beta	25.3574	0.8751	6.7435	45.6416
	MFO-Beta	25.3574	0.8751	6.7435	45.6416
	FPA-Beta	25.2979	0.8718	6.6265	47.3522
	SSA-Beta	25.3574	0.8751	6.7435	45.6416
	HE	11.8548	0.4589	6.5209	44.0052
	RETINEX	12.431	0.5842	5.5088	38.1079
Boat	VFOA-Beta	16.0082	0.9017	5.2517	19.8204
	FOA-Beta	10.4928	0.5394	4.1817	33.2120
	CSO-Beta	14.4093	0.6831	5.6887	20.5671
	MFO-Beta	14.4075	0.6825	5.6900	20.5692
	FPA-Beta	14.4089	0.6816	5.6892	20.5590
	SSA-Beta	14.4091	0.6821	5.6897	20.5643
	HE	17.7826	0.5480	4.7741	22.1750
	RETINEX	14.2996	0.6248	4.8725	23.3011
Soccer	VFOA-Beta	17.6745	0.7194	3.8923	32.0761
	FOA-Beta	15.7859	0.7310	3.8141	39.3886
	CSO-Beta	12.8125	0.6775	3.8900	34.8331
	MFO-Beta	12.8131	0.6769	3.8895	34.9527
	FPA-Beta	13.0340	0.6747	3.9498	35.0801
	SSA-Beta	12.8115	0.6770	3.8893	34.8470
	HE	14.6742	0.6217	3.5785	32.3902
	RETINEX	16.8695	0.9119	4.0198	31.5751
Flower	VFOA-Beta	17.9973	0.8827	2.6208	26.1631
	FOA-Beta	14.5428	0.7795	3.2147	31.0731
	CSO-Beta	14.2028	0.5661	2.4150	28.6446
	MFO-Beta	14.2020	0.5655	2.4147	28.6452
	FPA-Beta	14.2221	0.5645	2.5202	28.5278
	SSA-Beta	14.2023	0.5658	2.4144	28.6437
	HE	17.2677	0.3997	2.1978	27.0770
	RETINEX	18.3894	0.8062	2.3942	30.9134
Endoscopy1	VFOA-Beta	18.7242	0.9206	3.6254	35.6427
	FOA-Beta	13.4501	0.8345	4.2639	40.3675
	CSO-Beta	15.9342	0.7293	3.6246	39.1033
	MFO-Beta	15.8961	0.7405	3.6233	39.1220
	FPA-Beta	15.9045	0.7387	3.6241	39.1103
	SSA-Beta	15.9241	0.7390	3.6250	39.1176
	HE	12.7799	0.1446	3.5737	44.3810
	RETINEX	13.8180	0.6861	3.7670	41.1695
Endoscopy2	VFOA-Beta	20.6469	0.9485	3.4683	31.4070
	FOA-Beta	14.6084	0.8095	3.8795	47.3367
	CSO-Beta	14.0327	0.5501	3.5592	40.6031
	MFO-Beta	14.0262	0.5459	3.5607	40.5985
	FPA-Beta	14.0282	0.5498	3.4316	38.4062
	SSA-Beta	14.0262	0.5459	3.5607	40.5985
	HE	15.5600	0.2809	3.1787	29.0851
	RETINEX	14.9174	0.4878	3.7199	28.6450

## Data Availability

Not applicable.
